# Supplementary material for: mRNA trafficking directs cell-size-scaling of mitochondria distribution and function
Source: Nat Commun. 2025 Jul 31;16:7029. doi: 10.1038/s41467-025-61940-6 (PMC12313994; doi:10.1038/s41467-025-61940-6)

Title: mRNA trafficking directs cell-size-scaling of mitochondria distribution and function

Authors: Joshua J. Bradbury[1,2], Georgia E. Hulmes[1]†, Ranjith Viswanathan[1]†, Guilherme Costa[1,3], Holly E. Lovegrove[1], Shane P. Herbert[1]*

[1] Faculty of Biology Medicine and Health, Michael Smith Building, University of Manchester; Oxford Road, Manchester, UK.

[2] Centre for Developmental Neurobiology, New Hunt's House, King's College London, UK.

[3] School of Medicine, Dentistry and Biomedical Sciences, Wellcome Wolfson Institute for Experimental Medicine, Queen's University Belfast, UK.

* Corresponding author. E-mail: shane.herbert@manchester.ac.uk

† These authors contributed equally: Georgia E. Hulmes, Ranjith Viswanathan

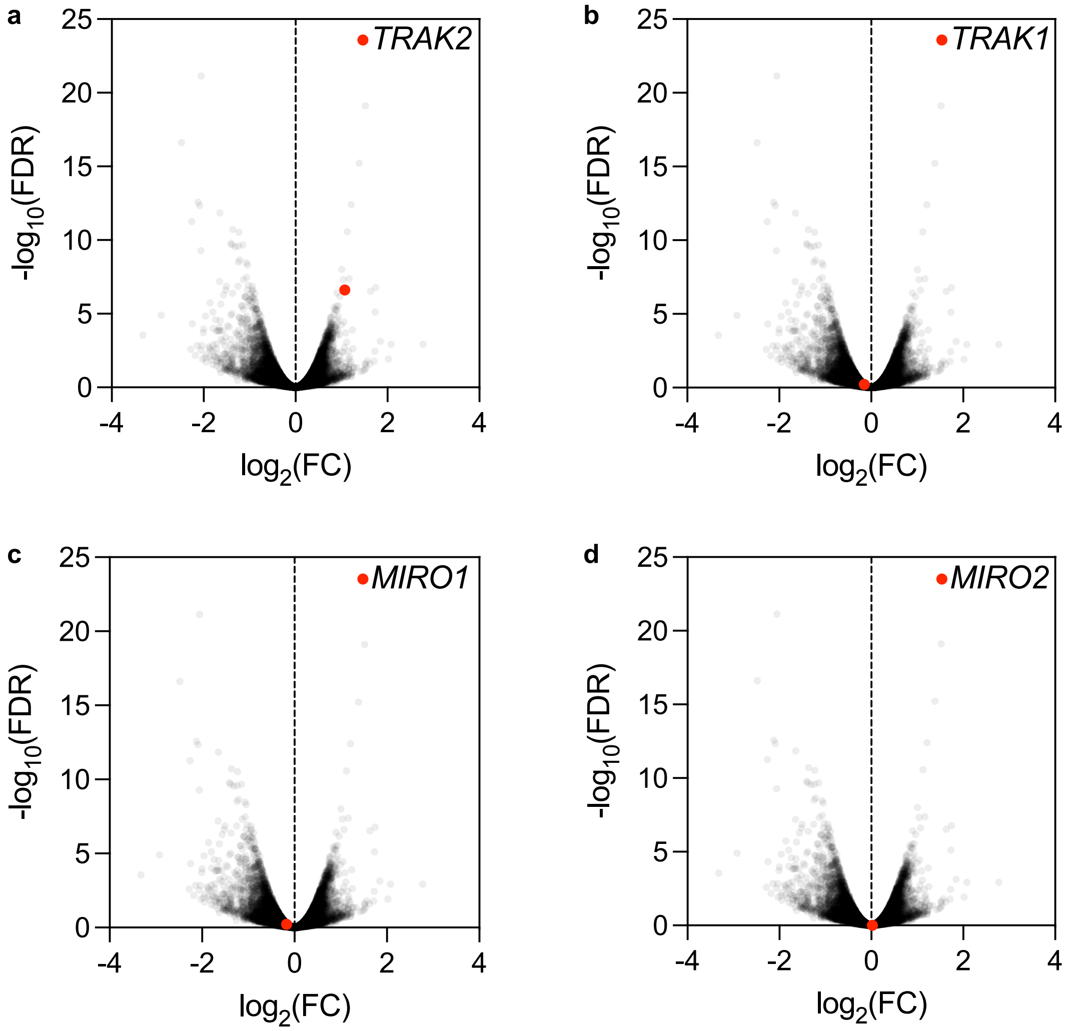
a
b
c
d
TRAK2
TRAK1
MIRO1
MIRO2
-log10(FDR)
log2(FC)
25
20
15
10
5
0
-4
-2
0
2
4

Supplementary Fig. 1: *TRAK2* mRNA is polarised in migrating endothelial cells.

a-**c**, Volcano plots of mRNAs differentially enriched in cell protrusions over cell bodies in migrating endothelial cells, as generated previously[1], with *TRAK2* (**a**), *TRAK1* (**b**), *MIRO1* (**c**) and *MIRO2* (**d**) indicated by red dots. RNAseq data are plotted in $\log_2$ fold change (FC) levels of protrusions over cell bodies against adjusted $-\log_{10}$ false discovery rate (FDR).

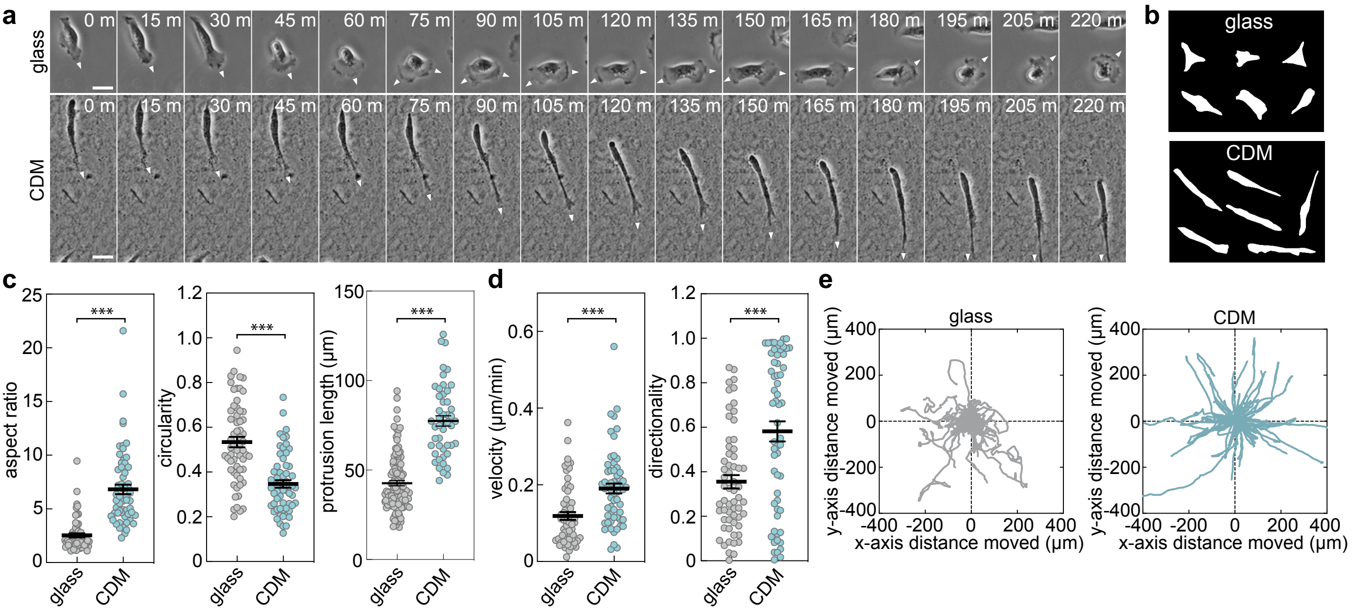

a
glass
CDM
0 m
15 m
30 m
45 m
60 m
75 m
90 m
105 m
120 m
135 m
150 m
165 m
180 m
195 m
205 m
220 m
b
glass
CDM
c
aspect ratio
circularity
protrusion length (μm)
***
glass
CDM
d
velocity (μm/min)
directionality
e
glass
CDM
y-axis distance moved (μm)
x-axis distance moved (μm)

Supplementary Fig. 2: Impact of substrate on cell morphology and behaviour.

a, Still images of migrating human endothelial cells (ECs) cultured either on glass or CDM (arrowheads indicate direction of motile cell protrusions). **b**, Representative examples of EC morphology when cultured on glass or CDM. **c**, Quantification of EC morphometrics (aspect ratio, circularity and protrusion length) when cultured either on glass or CDM (n=60 cells on glass for aspect ratio and circularity, n=121 cells on glass for protrusion length, n=61 cells on CDM for aspect ratio and circularity, n=48 cells on CDM for protrusion length, two-tailed Mann-Whitney test, ***P=<0.0001 for all). **d**, Quantification of EC velocity and directional persistence when cultured either on glass or CDM (n=60 cells glass, n=59 cells CDM, two-tailed Mann-Whitney test, ***P=<0.0001 for velocity, 0.0003 for directionality). **e**, Rose plots of motile cell trajectories when cultured either on glass or CDM (n=60 cells glass, n=59 cells CDM). Scale bars, 20μm.

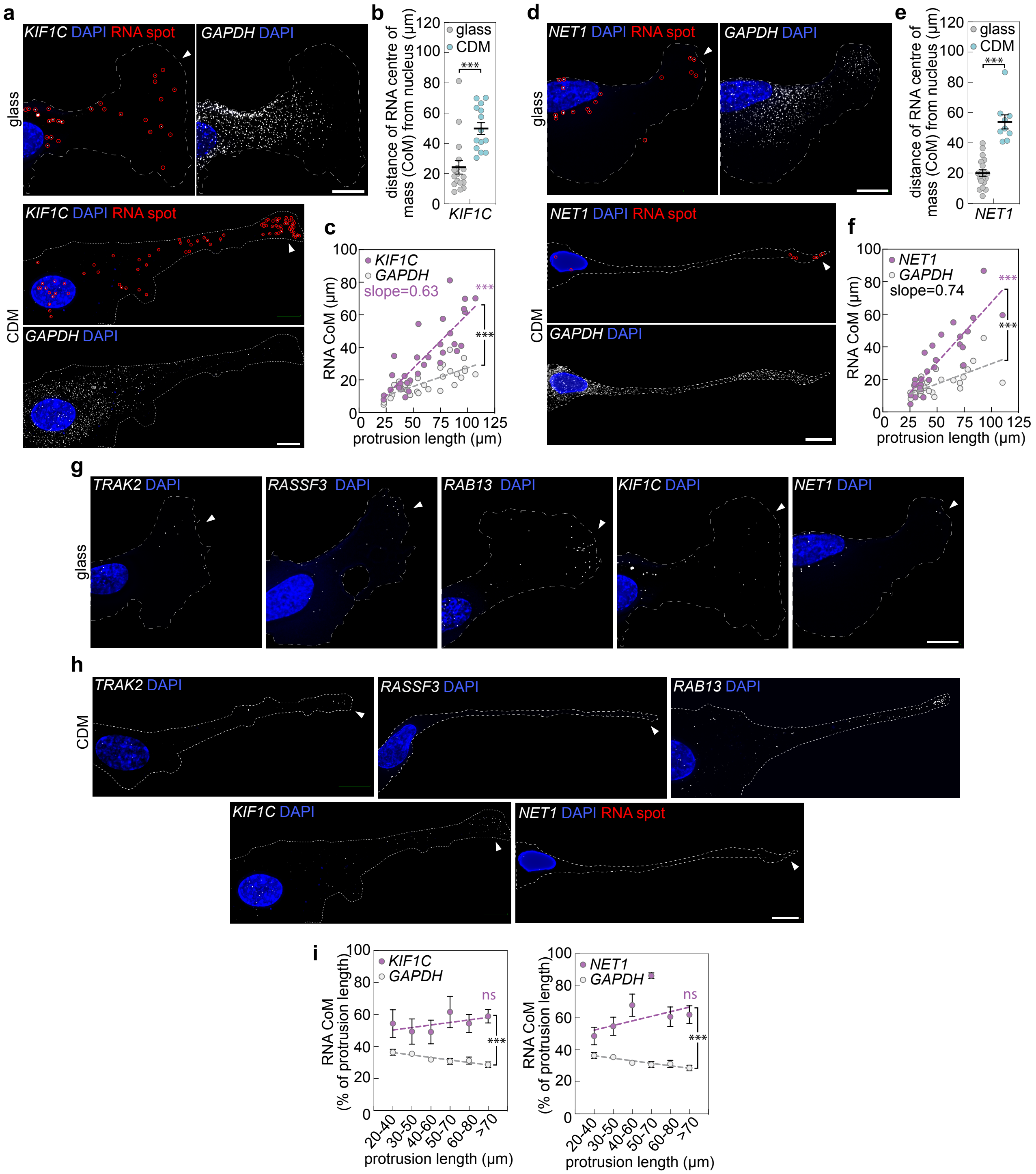
a
KIF1C DAPI RNA spot
GAPDH DAPI
glass
CDM
b
distance of RNA centre of mass (CoM) from nucleus (µm)
glass
CDM
***
KIF1C
c
KIF1C
GAPDH
slope=0.63
RNA CoM (µm)
protrusion length (µm)
d
NET1 DAPI RNA spot
e
NET1
f
NET1
slope=0.74
g
TRAK2 DAPI
RASSF3 DAPI
RAB13 DAPI
KIF1C DAPI
NET1 DAPI
h
i
RNA CoM (% of protrusion length)
ns
20-40
30-50
40-60
50-70
60-80
>70

Supplementary Fig. 3: Size-dependent targeting of *KIF1C* and *NET1* to cell protrusions. **a**,**d**, smFISH detection of *KIF1C*, and *NET1* mRNAs in exemplar ECs cultured either on glass or CDM (red circles indicate distinct mRNA spots; arrowheads indicate mRNA accumulation at distal sites in protrusions; dashed line indicates cell outline). **b**,**e**, Quantification of the distance that the RNA centre of mass (CoM) sits from the nucleus for the indicated mRNAs when ECs were cultured either on glass or CDM (*n*=17 cells glass *KIF1C*, *n*=14 cells CDM *KIF1C*, *n*=19 cells glass *NET1*, *n*=19 cells CDM *NET1*, two-tailed Mann-Whitney test, ****P*=<0.0001 for all). **c**,**f**, Plots comparing the distance that the RNA CoM sits from the EC nucleus versus protrusion length for the indicated mRNAs (*n*=32 cells for *KIF1C*, *n*=28 cells for *NET1*; two-tailed Pearson's correlation coefficient, magenta asterisks, ****P*=<0.0001 for all; analysis of covariance, black asterisks, ****P*=0.0006 versus *GAPDH* for *KIF1C*, <0.0001 versus *GAPDH* for *NET1*). **g**,**h**, smFISH detection of *TRAK2, RASSF3, RAB13, KIF1C*, and *NET1* mRNAs in exemplar ECs (from Fig.1a,d,g and Supplementary Fig.3a,d) cultured either on glass (**g**) or CDM (**h**) without the labelling of RNA spots with red circles (arrowheads indicate mRNA accumulation at distal sites in protrusions; dashed line indicates cell outline). **i**, Plots comparing the distance that the RNA CoM sits from the EC nucleus normalised to protrusion length versus protrusion length for the indicated mRNAs (*n*=as in **c**,**f**; two-tailed Pearson's correlation coefficient, magenta asterisks, ns *P*=0.2757 for *KIF1C*, 0.4061 for *NET1*; analysis of covariance, black asterisks, ****P*=0.0038 versus *GAPDH* for *KIF1C*, <0.0001 versus *GAPDH* for *NET1*). For analyses in **c**,**f**,**i**, data for cells cultured on glass and CDM were pooled. Data are mean ± s.e.m. (**b**,**e**,**i**). For **a**,**d**,**g**,**h** scale bars, 10μm.

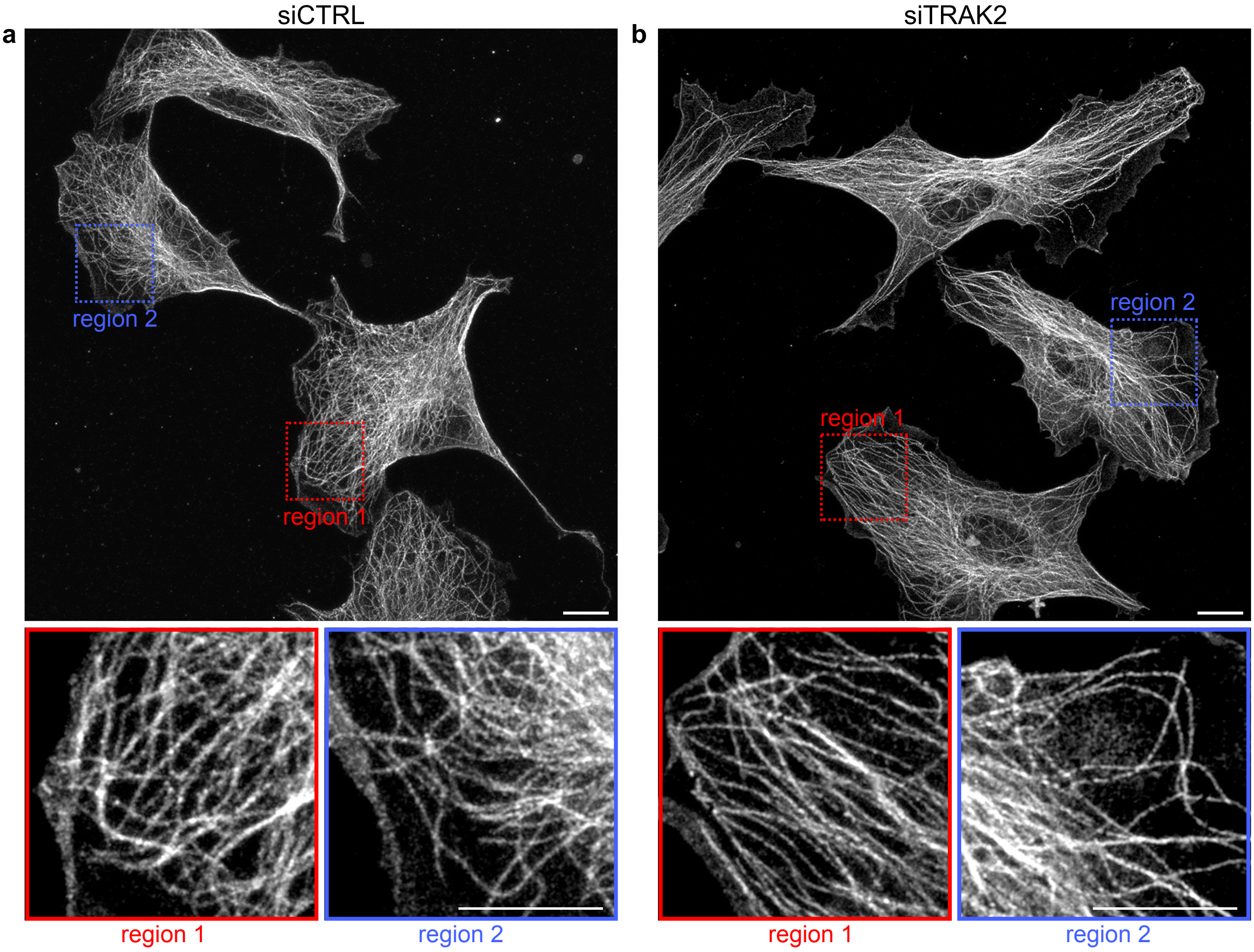
a
siCTRL
region 2
region 1
region 1
region 2
b
siTRAK2
region 2
region 1
region 1
region 2

Supplementary Fig. 4: *TRAK2* knockdown does not perturb the microtubule cytoskeleton. **a**,**b**, Representative images of alpha-tubulin-immunostained siCTRL (**a**) or siTRAK2 (**b**) treated ECs (red and blue boxes indicate the regions magnified below). *n*=3 independent experiments. Scale bars, 10μm.

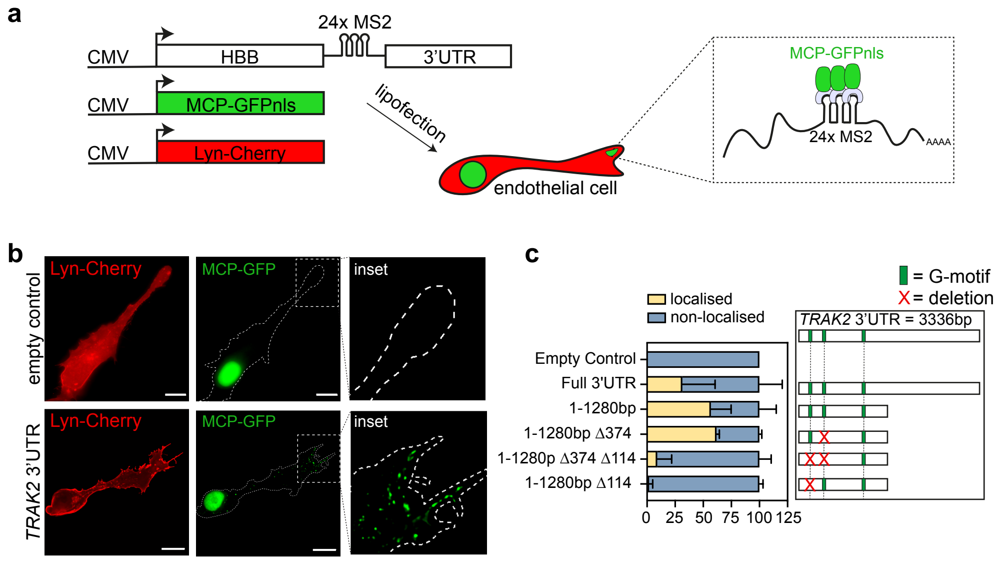
a
CMV
HBB
24x MS2
3'UTR
CMV
MCP-GFPnls
CMV
Lyn-Cherry
lipofection
endothelial cell
MCP-GFPnls
24x MS2
AAAA
b
empty control
TRAK2 3'UTR
Lyn-Cherry
MCP-GFP
inset
c
= G-motif
= deletion
localised
non-localised
TRAK2 3'UTR = 3336bp
Empty Control
Full 3'UTR
1-1280bp
1-1280bp Δ374
1-1280p Δ374 Δ114
1-1280bp Δ114
0 25 50 75 100 125

Supplementary Fig. 5: A single 3'UTR G-motif drives targeting of *TRAK2* mRNA.

a, Schematic showing the *in vitro* MS2 system strategy used to identify 3'UTR elements driving mRNA localisation to protrusions. CMV promoter induced expression of lyn-Cherry, MCP-GFPnls, and hHBB-24xMS2-tagged 3′UTRs enables MCP-GFPnls to be used to visualise the localisation of 24xMS2-tagged 3'UTRs. **b**, Representative cells co-expressing lyn-Cherry, MCP-GFPnls, and either 24xMS2 or 24xMS2-*TRAK2* 3'UTR (dashed line indicates cell outline). **c**, Percentage of cells with MCP-GFPnls located in protrusions when transfected with either Wt *TRAK2* 3'UTR or the truncations and deletions of the *TRAK2* 3'UTR indicated in the panel to the right (*n*=3 independent experiments). This work is an extension of previous analyses[1]. Data are mean ± s.e.m. (**c**). Scale bars, 10μm.

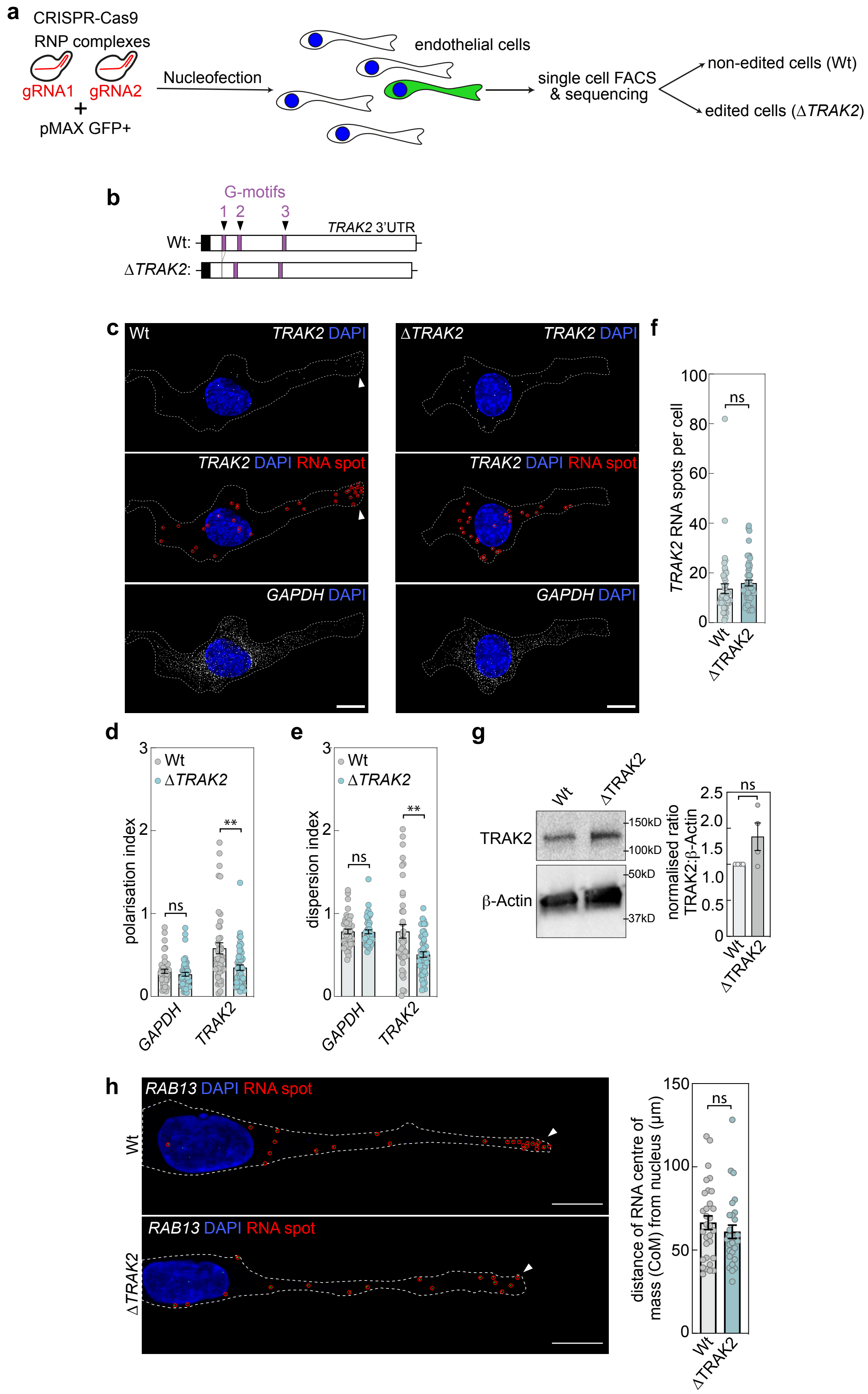
a
CRISPR-Cas9
RNP complexes
gRNA1
gRNA2
+
pMAX GFP+
Nucleofection
endothelial cells
single cell FACS
& sequencing
non-edited cells (Wt)
edited cells (ΔTRAK2)
b
G-motifs
1 2 3
TRAK2 3'UTR
Wt:
ΔTRAK2:
c
Wt
TRAK2 DAPI
TRAK2 DAPI RNA spot
GAPDH DAPI
ΔTRAK2
f
TRAK2 RNA spots per cell
ns
d
polarisation index
Wt
ΔTRAK2
ns
**
GAPDH
TRAK2
e
dispersion index
g
TRAK2
β-Actin
150kD
100kD
50kD
37kD
normalised ratio TRAK2:β-Actin
h
RAB13 DAPI RNA spot
distance of RNA centre of mass (CoM) from nucleus (µm)

Supplementary Fig. 6: Deletion of the 24bp motif perturbs targeting of *TRAK2* mRNA.

a, Schematic showing the strategy used to generate EC lines with both an excised 3'UTR localisation element (*ΔTRAK2*) and a non-edited control EC line (Wt). **b**, Illustration of removal of the 24bp G-motif from the endogenous *TRAK2* 3'UTR to generate *ΔTRAK2* (Black, white and magenta regions indicate the final exon of *TRAK2*, 3'UTRs and G-motifs, respectively). **c**, smFISH co-detection of *TRAK2* and *GAPDH* mRNAs in exemplar Wt or *ΔTRAK2* ECs cultured on glass (red circles indicate distinct mRNA spots; arrowheads indicate mRNA accumulation at distal sites in protrusions in Wt cells; dashed line indicates cell outline as defined by F-actin labelling). **d**,**e**, Quantification of the polarisation index (**d**) and dispersion index (**e**) of *TRAK2* and *GAPDH* mRNAs in Wt and *ΔTRAK2* ECs cultured on glass (*n*=44 cells Wt polarisation index, *n*=51 *ΔTRAK2* cells *GAPDH* polarisation index, *n*=52 *ΔTRAK2* cells *TRAK2* polarisation index, *n*=43 cells Wt dispersion index *GAPDH*, *n*=41 cells Wt dispersion index *TRAK2*, *n*=50 *ΔTRAK2* cells *GAPDH* dispersion index, *n*=52 *ΔTRAK2* cells *TRAK2* dispersion index, two-tailed Mann-Whitney test, ***P*=0.0035 for polarisation index, 0.0096 for dispersion index, ns *P*=0.2373 for polarisation index, 0.9526 for dispersion index). **f**, Quantification of the number of *TRAK2* mRNA spots detected in Wt and *ΔTRAK2* ECs cultured on glass (*n*=44 cells Wt, *n*=52 cells *ΔTRAK2*, two-tailed unpaired t-test, ns *P*=0.3119). **g**, Representative Western blot for TRAK2 and β-Actin in Wt and *ΔTRAK2* ECs and densitometric analysis the ratio of TRAK2:β-Actin levels (*n*=4 independent experiments, two-tailed Mann-Whitney test, ns *P*=0.3143). **h**, smFISH detection of *RAB13* mRNAs in exemplar Wt or *ΔTRAK2* ECs cultured on CDM (red circles indicate distinct mRNA spots; arrowheads indicate mRNA accumulation at distal sites in protrusions; dashed line indicates cell outline as defined by F-actin labelling) and quantification of the RNA CoM (*n*=31 cells Wt, *n*=28 cells *ΔTRAK2*, two-tailed Mann-Whitney test, ns *P*=0.3282). Data are mean ± s.e.m. (**d**,**e**,**f**,**g**,**h**). For **c** and **h** scale bars, 10µm.

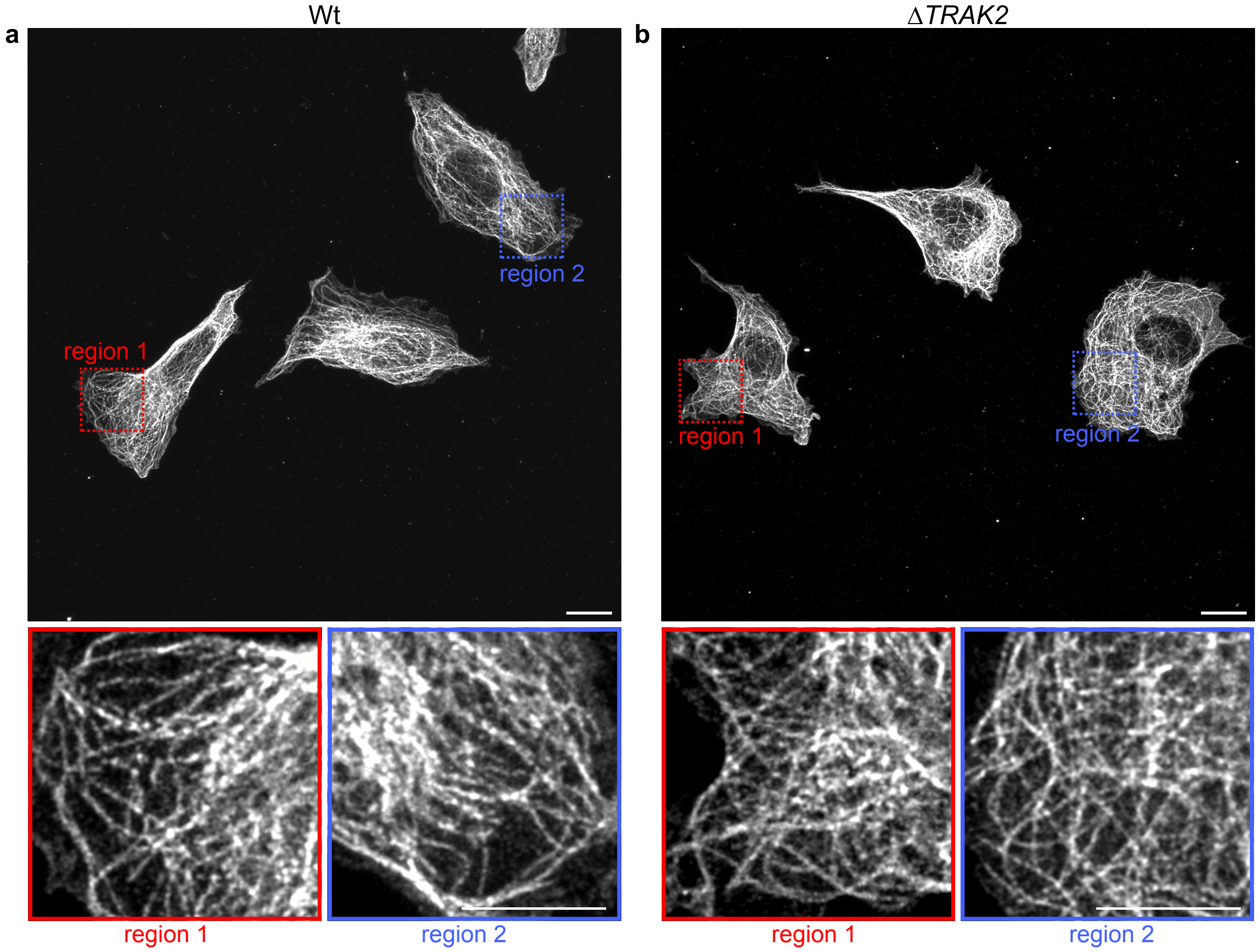
a
Wt
region 1
region 2
b
ΔTRAK2
region 1
region 2
region 1
region 2
region 1
region 2

Supplementary Fig. 7: Loss*TRAK2* mRNA targeting does not perturb the microtubule cytoskeleton. **a**,**b**, Representative images of alpha-tubulin-immunostained Wt (**a**) or *ΔTRAK2* (**b**) ECs (red and blue boxes indicate the regions magnified below). *n*=3 independent experiments. Scale bars, 10μm.

Supplementary table 1: Oligonucleotide Sequences.

**CRISPr/Cas9**	
**Name**	**Sequences (5' – 3')**
5' crRNA *TRAK2* 3' UTR	AGAAAAGGAATGTTGCACAA
3' crRNA *TRAK2* 3' UTR	GGAGGAATGGAAATAAAATT
**Genotyping**	
**Name**	**Sequences (5' – 3')**
TRAK2 3' UTR F	TGAAACATGTGGTCTGGTCTG
TRAK2 3' UTR R	TCCCTCTGAACACTCATGGC
**Cloning**	
**Name**	**Sequences (5' – 3')**
NheI 1nt *TRAK2* 3'UTR F	GGAAGCTAGCGGTTCAGCAGTTAACTGACC
XhoI 3336nt *TRAK2* 3'UTR R	GGAACTCGAGTGAAAATTCAAGCTACTCATG
XhoI 1280nt *TRAK2* 3'UTR R	GGAACTCGAGATCTAACCCCAGAGCCTCAC
del114-142 *TRAK2* R	GGGCTCATCCCAATTTTATCACAACCCTTGTGCAACAT
del114-142 *TRAK2 F*	ATGTTGCACAAGGGTTGTGATAAAATTGGGATGAGCCC
del374-402 *TRAK2* F	AAGCATAAAGCAGAGAGAACCCCAGTTTTATTGCTTATAGAAAGC
del374-402 *TRAK2* R	GCTTTCTATAAGCAATAAAACTGGGGTTCTCTCTGCTTTATGCTT
**Notes:** underlined uppercase - restriction site	

Supplementary table 2: Probe Sequences.

mRNA	Probe sequence (5' - 3')		mRNA	Probe sequence (5' - 3')		mRNA	Probe sequence (5' - 3')
ACTB	gctcgagccataaaaggcaa		*KIF1C*	tcttctatcacctactcgg		*NET1*	gccgagtcagaaccaaga
ACTB	cgatatcatcatccatggtg		*KIF1C*	ttggtttgctcctggcaac		*NET1*	ccgttcgttccgtgtgac
ACTB	cacgatggaggggaagacgg		*KIF1C*	cttcctatctctcttca		*NET1*	gccggtaaacctggtaag
ACTB	acataggaatccttctgacc		*KIF1C*	cagcgtttgtgggacacac		*NET1*	ctcttggactgggattgg
ACTB	ggtacttcagggtgaggatg		*KIF1C*	gtcaaacactggctacgca		*NET1*	tgcaggtcttctaggact
ACTB	cagattttctccatgtcgtc		*KIF1C*	acttatgatcctaccatgc		*NET1*	ccattctcacatctccat
ACTB	acacgcagctcattgtagaa		*KIF1C*	ccaaatatagcaggaggcc		*NET1*	agctcctcgaaaggagcc
ACTB	acatgatctgggtcatcttc		*KIF1C*	cagccaaaccagtcaggtc		*NET1*	gctttctctgagttactg
ACTB	ggatagcacagcctggatag		*KIF1C*	tattaaagggcccacatcc		*NET1*	gaggggtcatggaagcga
ACTB	catcacgatgccagtggtac		*KIF1C*	aagctccctgtttgctatc		*NET1*	gagtgtgagactgggctg
ACTB	tcgtagatgggcacagtgtg		*KIF1C*	aaagatcgggctgcgcacc		*NET1*	gtggaacacgtcattggc
ACTB	tcttcatgaggtagtcagtc		*KIF1C*	cttctgtgctttgtccata		*NET1*	cagttgaaccactgctgc
ACTB	taatgtcacgcacgatttcc		*KIF1C*	aagcattgacaggctggcg		*NET1*	cgactggaagggggcaat
ACTB	atctcttgctcgaagtccag		*KIF1C*	atctgtcagcaacagccag		*NET1*	tgcagctcaggtggactg
ACTB	cagggaggagctggaagcag		*KIF1C*	gtaccccacaaatccttct		*NET1*	tcacactcttcgtgcagc
ACTB	tcattgccaatggtgatgac		*KIF1C*	gacttaaagcagaactcct		*NET1*	tgagtttcctcgcagagg
ACTB	gaaggtagtttcgtggatgc		*KIF1C*	gcttgggagacctttcaag		*NET1*	gaaactgtggatgccctc
ACTB	cgtcacacttcatgatggag		*KIF1C*	aggtaagcgcagtatgctt		*NET1*	gcgttttcatcaacttct
ACTB	tacaggtctttgcggatgtc		*KIF1C*	gcaggtaacacaaccagga		*NET1*	atgccagagccacatctg
ACTB	caatgccagggtacatggtg		*KIF1C*	aacaatgatgtagcctgcc		*NET1*	agctcttgctgtcctctg
ACTB	atcttcattgtgctgggtgc		*KIF1C*	gcctcaagccagaacatta		*NET1*	ctgtgtctggtgtgtctt
ACTB	ctcaggaggagcaatgatct		*KIF1C*	tggcatagtcaagggctcg		*NET1*	tccctcgctcttcggatg
ACTB	cgatccacacggagtacttg		*KIF1C*	acctgcaaaatcctggcaa		*NET1*	tgccaccagaaagggctt
ACTB	tcatactcctgcttgctgat		*KIF1C*	gcagtgctgtagtacaatc		*NET1*	caccaaagtctctttccg
ACTB	atttgcggtggacgatggag		*KIF1C*	ccagagtctggcaagccaa		*NET1*	aacacacagagccttctc
ACTB	aagtcatagtccgcctagaa		*KIF1C*	aagcagtagctcagagctt		*NET1*	gtaaccctatgatgctcc
ACTB	gtcaagaaagggtgtaacgc		*KIF1C*	ctatggttttggctggaga		*NET1*	taccaggcttaccatgtt
ACTB	ttttctgcgcaagttaggtt		*KIF1C*	aacctaatcgttccttcgc		*NET1*	agccatgatgaatccaga
ACTB	cattgtgaactttgggggat		*KIF1C*	aaaggcaggagcatcaggg		*NET1*	actggcagaattccagca
ACTB	gtgcaatcaaagtcctcggc		*KIF1C*	ggcagtcagaggtgtcaaa		*NET1*	aggttttgggtgccaatg
ACTB	cctgtaacaacgcatctcat		*KIF1C*	ctgtcaacaggtctgtgaa		*NET1*	ctccagacatacattcca
ACTB	cttttaggatggcaagggac		*KIF1C*	tacattccagatcttgagt		*NET1*	tggtttgttttccaagga
ACTB	tctccttagagagaagtggg		*KIF1C*	cactgttgccaaaccatcc		*NET1*	taaaaacctcctcaggct
ACTB	gtggacttgggagaggactg		*KIF1C*	tctatcattttcttgctca		*NET1*	ggcttggtttcacttggt
ACTB	aaagcaatgctatcacctcc		*KIF1C*	gcattctcggtaacactca		*NET1*	ccctgaaatgctacactt
ACTB	catacatctcaagttggggg		*KIF1C*	ttttctcatctcaaacccc		*NET1*	agccatttcatgggtctt
ACTB	actcccagggagaccaaaag		*KIF1C*	tctaagtaaagtttgccca		*NET1*	ccctcttgcatttcagac
ACTB	gtctcaagtcagtgtacagg		*KIF1C*	cttgatggcctagaggggt		*NET1*	ccccctttcaaagatgat
ACTB	ggtgtgcacttttattcaac		*KIF1C*	gaggtgatggtgatgtctg		*NET1*	atcgggcttttactcctc
			KIF1C	aaggccccatggattctaa		*NET1*	ttatcatttccactggcc

mRNA	Probe sequence (5' - 3')		mRNA	Probe sequence (5' - 3')		mRNA	Probe sequence (5' - 3')
RAB13	caggccaggaagaagttttc		*RASSF3*	ggtgaagaagaagtcctcgg		*TRAK2*	cacacaattggggaagccaa
RAB13	tggacggttggcaaacagag		*RASSF3*	ttcaacttgtctgtgactgc		*TRAK2*	ctttggacagtcatgactgg
RAB13	cagcaacttgaagaggtggt		*RASSF3*	cccatttgaattcaaggtca		*TRAK2*	gcactctcatgtgattatca
RAB13	atgatcagacaagtcttgcc		*RASSF3*	cagagttccatctgtacttt		*TRAK2*	gtcaaatgacatccgtagca
RAB13	gttgttgaagttgtcctctg		*RASSF3*	tggagaagtctgtggaggtt		*TRAK2*	aaatggatttggtcacatgg
RAB13	ttccgatggtggagatgtaa		*RASSF3*	tgggagagagttttccagaa		*TRAK2*	ggaaatccatcaagccattc
RAB13	ccacagtgcggatcttgaaa		*RASSF3*	tgtattcatacagccattgc		*TRAK2*	gctttggtatgaatcagagt
RAB13	ccagacttgtagtttgatct		*RASSF3*	tgtttgtgctgctgatatga		*TRAK2*	ctttcttgctttggttgaga
RAB13	attgtcttgaaccgctcttg		*RASSF3*	gctctcagtcacgagaaact		*TRAK2*	attctgggattgactcatgc
RAB13	tccacggtagtaggcagtag		*RASSF3*	ccctgtgacaacgcttataa		*TRAK2*	ttcacctgttggtgatgtaa
RAB13	atactaggataatgcccatg		*RASSF3*	taccaaacgcaggtagagtg		*TRAK2*	gagtctctgtgattgctatt
RAB13	gatttctcatccgtgatgtc		*RASSF3*	actaagtgtgtctgttctgg		*TRAK2*	cattggagcagacatcagtg
RAB13	ccagttctgaatattctcga		*RASSF3*	ctccaatttcatgttcacga		*TRAK2*	agctcaacttcagggagatc
RAB13	cattctccttgatgcttttc		*RASSF3*	tagttctggaaggctgaagg		*TRAK2*	gtagttgttcttctagcaga
RAB13	ctccatgtcacatttgttcc		*RASSF3*	ttgtccaagatgcgcaagaa		*TRAK2*	agtgtctacttttagcctat
RAB13	aaatcggattccatgctctc		*RASSF3*	ctttaatcaggcttccacac		*TRAK2*	agtccagtcttgattttcat
RAB13	ctggatttagcactagtttc		*RASSF3*	ttgctgctgttttgtacatc		*TRAK2*	aacggaaagtctcttcagca
RAB13	aaaagcctcatccacattca		*RASSF3*	gatagaaaggcgaagaccct		*TRAK2*	ctgtctgtgcctagaatcat
RAB13	ctcctgacttgagcaagatg		*RASSF3*	gaccaggtttggggaacaaa		*TRAK2*	gtaagttttggtcatctgct
RAB13	agttttcaggtcagtactgg		*RASSF3*	ttacatgggacgctaaggtg		*TRAK2*	ggagatgtgtaaccatgtcg
RAB13	acttgttggtgttcttcttg		*RASSF3*	gtgctgaacaacaagcttgc		*TRAK2*	cagatcacgatccctctctg
RAB13	aggcaagaaagggtcctcag		*RASSF3*	cttgccaagaggtaacactg		*TRAK2*	tgtccaattcgagcagcgag
RAB13	tacctatgtgaccctccaag		*RASSF3*	cctttcgattcacatggata		*TRAK2*	acatggttccgctttaagag
RAB13	aacccaggtaaggtctgaag		*RASSF3*	aattcctcagagttcctaga		*TRAK2*	ggattcgttctgctcagata
RAB13	tttacatttatgtttgccct		*RASSF3*	actgaaaagccaagtcacct		*TRAK2*	tcaaaggcttgtcccaattg
RAB13	ggaccctaaaacctgatcta		*RASSF3*	aatgggtttaacacctccac		*TRAK2*	atgctgcagctgattaactt
RAB13	gagcaaattccctagtgtag		*RASSF3*	atattctacccaccaggaaa		*TRAK2*	actcatctttcttgcatagc
RAB13	agaccatgacaagtgacaga		*RASSF3*	gggcagaattaaagggtcgg		*TRAK2*	cttcagaagcaatggagacg
RAB13	tgcaaatggtggcctttaat		*RASSF3*	aatagccctgttctctatta		*TRAK2*	cagctggaatcagtttcact
			RASSF3	tggtaggattgggtctttat		*TRAK2*	tcattgaaccgaagaggtgt
			RASSF3	ttctattgcacatgcatctc		*TRAK2*	gcaacccttgagataagcta
			RASSF3	gaggttatttagcctgtttt		*TRAK2*	ttttcttgcagcatttccaa
			RASSF3	ccctctgttctcagattaaa		*TRAK2*	catattctcttcttccagtt
			RASSF3	aatccattaggtgtcaccaa		*TRAK2*	gacaagccttggatcgaaga
			RASSF3	ctgaagcaccttggaatttc		*TRAK2*	aggtaacagtttctgtcttt
			RASSF3	aaaggcacagttcacttggg		*TRAK2*	agctgttgttccttttcttc
			RASSF3	cagaagagccaccaagagac		*TRAK2*	ttctttaacacagtcgctga
			RASSF3	ttaagtctgcctccaacaag		*TRAK2*	tctgagcatttgtttcacga
			RASSF3	gtaaattgtgtggcacagca		*TRAK2*	gaatcagctcatcactcttc
			RASSF3	gaaggcattccctgaacaat		*TRAK2*	agaggaaagctcttcttggt
			RASSF3	atactcaaatccaggccata		*TRAK2*	aggtctacaatctgtgacaa
			RASSF3	ccatcagctactactttagg		*TRAK2*	atgttctttaagtttgtgct
			RASSF3	gatgaggatgcctattcaga		*TRAK2*	catctttggaagcttgcagg
			RASSF3	cacatgacagtaagcgaggt		*TRAK2*	ctgtcttgtaactcgtgcag
			RASSF3	accagtgtttgggaaaggac		*TRAK2*	aacattcctagacactccat
			RASSF3	aataccaccccttcatttaa		*TRAK2*	gggccagatctactacgaag
			RASSF3	tgccatttaaaatgctctcc		*TRAK2*	cccagtaaaagctccatatg
						TRAK2	caatctcagctgccaaagat

Uncropped Scan of blot from Supplementary Fig. 6g

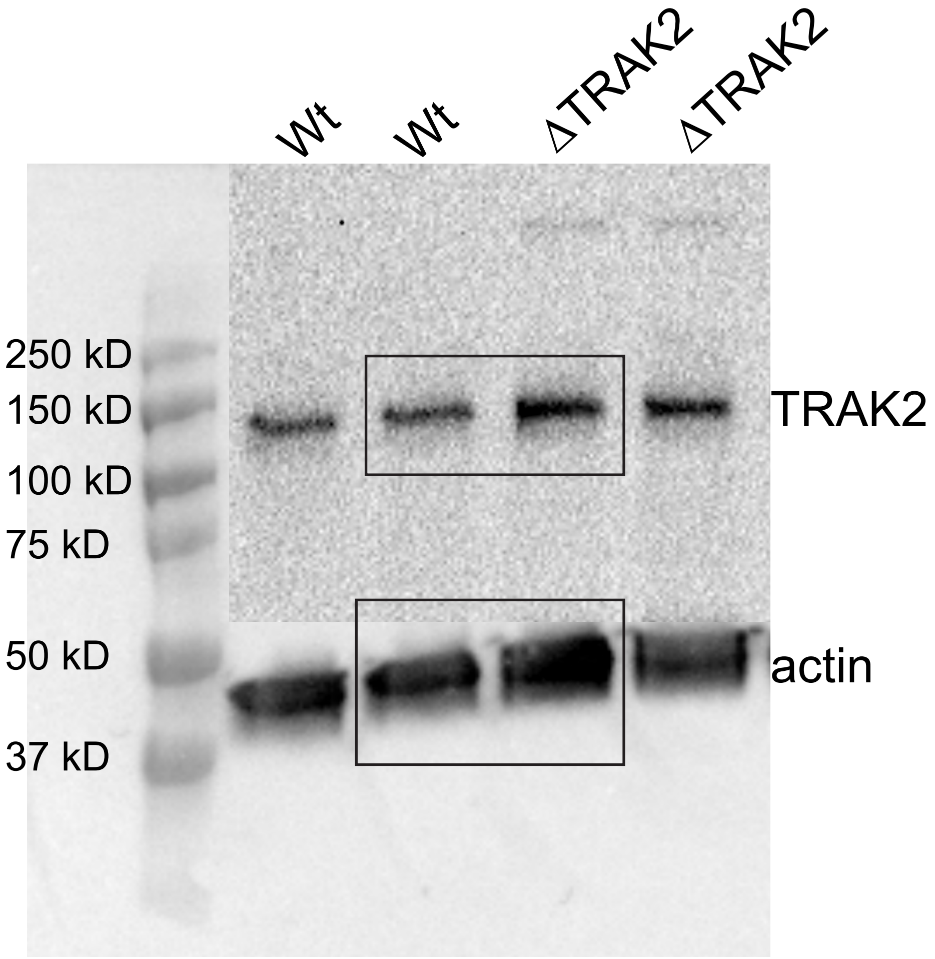

Supplement: Supplementary file 1 — Supplementary Information [file 41467_2025_61940_MOESM1_ESM.pdf]
