# Supplementary material for: mRNA trafficking directs cell-size-scaling of mitochondria distribution and function
Source: Nat Commun. 2025 Jul 31;16:7029. doi: 10.1038/s41467-025-61940-6 (PMC12313994; doi:10.1038/s41467-025-61940-6)
Supplement: Supplementary file 2 — Reporting Summary [file 41467_2025_61940_MOESM2_ESM.pdf]

nature portfolio

Corresponding author(s): Shane P Herbert

Last updated by author(s): Apr 21, 2025

Reporting Summary

Nature Portfolio wishes to improve the reproducibility of the work that we publish. This form provides structure for consistency and transparency in reporting. For further information on Nature Portfolio policies, see our Editorial Policies and the Editorial Policy Checklist.

Statistics

For all statistical analyses, confirm that the following items are present in the figure legend, table legend, main text, or Methods section.

n/a	Confirmed	
☐	☒	The exact sample size (n) for each experimental group/condition, given as a discrete number and unit of measurement
☐	☒	A statement on whether measurements were taken from distinct samples or whether the same sample was measured repeatedly
☐	☒	The statistical test(s) used AND whether they are one- or two-sided<br>*Only common tests should be described solely by name; describe more complex techniques in the Methods section.*
☐	☒	A description of all covariates tested
☒	☐	A description of any assumptions or corrections, such as tests of normality and adjustment for multiple comparisons
☐	☒	A full description of the statistical parameters including central tendency (e.g. means) or other basic estimates (e.g. regression coefficient) AND variation (e.g. standard deviation) or associated estimates of uncertainty (e.g. confidence intervals)
☐	☒	For null hypothesis testing, the test statistic (e.g. F, t, r) with confidence intervals, effect sizes, degrees of freedom and P value noted<br>*Give P values as exact values whenever suitable.*
☒	☐	For Bayesian analysis, information on the choice of priors and Markov chain Monte Carlo settings
☒	☐	For hierarchical and complex designs, identification of the appropriate level for tests and full reporting of outcomes
☐	☒	Estimates of effect sizes (e.g. Cohen's d, Pearson's r), indicating how they were calculated

Our web collection on statistics for biologists contains articles on many of the points above.

Software and code

Policy information about availability of computer code

Data collection	Imaging data were collected and processed using Carl Zeiss Zen Blue 3.3
Data analysis	Imaging data were analysed using FIJI (2.14.0/1,54j) and graphpad Prism 10 (v.10.23(347)).

For manuscripts utilizing custom algorithms or software that are central to the research but not yet described in published literature, software must be made available to editors and reviewers. We strongly encourage code deposition in a community repository (e.g. GitHub). See the Nature Portfolio guidelines for submitting code & software for further information.

Data

Policy information about availability of data

All manuscripts must include a data availability statement. This statement should provide the following information, where applicable:

- Accession codes, unique identifiers, or web links for publicly available datasets
- A description of any restrictions on data availability
- For clinical datasets or third party data, please ensure that the statement adheres to our policy

All data supporting the findings of this study are available within the paper, its Supplementary Information and Source Data.

Research involving human participants, their data, or biological material

Policy information about studies with human participants or human data. See also policy information about sex, gender (identity/presentation), and sexual orientation and race, ethnicity and racism.

Reporting on sex and gender	This study did not involve human research participants, their data, or biological material.
Reporting on race, ethnicity, or other socially relevant groupings	This study did not involve human research participants, their data, or biological material.
Population characteristics	This study did not involve human research participants, their data, or biological material.
Recruitment	This study did not involve human research participants, their data, or biological material.
Ethics oversight	This study did not involve human research participants, their data, or biological material.

Note that full information on the approval of the study protocol must also be provided in the manuscript.

Field-specific reporting

Please select the one below that is the best fit for your research. If you are not sure, read the appropriate sections before making your selection.

☒ Life sciences ☐ Behavioural & social sciences ☐ Ecological, evolutionary & environmental sciences

For a reference copy of the document with all sections, see nature.com/documents/nr-reporting-summary-flat.pdf

Life sciences study design

All studies must disclose on these points even when the disclosure is negative.

Sample size	Sample size was determined based on knowledge of previous published and unpublished experiments using similar reagents and protocols.
Data exclusions	No data was excluded from the analyses.
Replication	Experiments were repeated at least two times in order to ensure reproducibility. Attempts at replication were successful.
Randomization	All cultured cells were selected randomly for experimental processing, imaging and analysis.
Blinding	Blinding was precluded in this study due the nature of sample preparation and to ensure the correct collection of sample groups. As such, researchers were aware of sample identity. Standardized procedures were applied to all sample groups.

Reporting for specific materials, systems and methods

We require information from authors about some types of materials, experimental systems and methods used in many studies. Here, indicate whether each material, system or method listed is relevant to your study. If you are not sure if a list item applies to your research, read the appropriate section before selecting a response.

Materials & experimental systems

n/a	Involved in the study	
☐	☒	Antibodies
☐	☒	Eukaryotic cell lines
☒	☐	Palaeontology and archaeology
☒	☐	Animals and other organisms
☒	☐	Clinical data
☒	☐	Dual use research of concern
☒	☐	Plants

Methods

n/a	Involved in the study	
☒	☐	ChIP-seq
☐	☒	Flow cytometry
☒	☐	MRI-based neuroimaging

Antibodies

Antibodies used	rabbit anti-TRAK2 (1:1000 Western blot, Proteintech, 13770-1)<br>mouse anti-TRAK2 (1:500 Western blot, Thermo Fisher Scientific, MA5-27606)<br>mouse anti-MIRO1 (1:200 Western blot, proximity ligation assay, Abcam, CL1083)

April 2023

rabbit anti-β-actin (1:1000 Western blot, Cell Signalling technology, 4967)
rabbit polyclonal anti-TRAK2 (1:50 Immunoprecipitation, 1:200 proximity ligation assay, Thermo Fisher Scientific, PA5-34889)
mouse anti-alpha tubulin (1:100 Abcam ab7291)
rabbit IgG (1:50 Immunoprecipitation, Proteintech, 30000-0-AP)
goat anti-mouse AlexaFluor 568 (1:500 Immunofluorescence, Thermo Fisher Scientific, A-21043),
goat anti-rabbit AlexaFluor 488 (1:500 Immunofluorescence, Thermo Fisher Scientific, A-11008)

Validation

All of the primary antibodies used have been validated by the manufacturer and have been extensively utilised in the literature:

rabbit anti-TRAK2: https://www.ptglab.com/products/TRAK2-Antibody-13770-1-AP.htm?srsltid=AfmBOopiDfOfYwyDDndxCkkbpbc_bS3XVmByRGwKOKeK41DxVbzY7Ksz

mouse anti-TRAK2: https://www.thermofisher.com/antibody/product/TRAK2-Antibody-clone-S390-43-Monoclonal/MA5-27606

mouse anti-MIRO1: https://www.abcam.com/en-us/products/primary-antibodies/miro1-antibody-cl1083-ab188029?srsltid=AfmBOooC6j9yo7SRPnNTowBF7uhf5yWy-nSg9YUTQc7bHK3OIcQzkFc_

rabbit anti-β-actin: https://www.cellsignal.com/products/primary-antibodies/b-actin-antibody/4967?srsltid=AfmBOorDCn3jxvvz2tl9GZAaL3vOuMwIjle9VT4fdS7IBvDyTQSW5qjb

rabbit polyclonal anti-TRAK2: https://www.thermofisher.com/antibody/product/TRAK2-Antibody-Polyclonal/PA5-34889

mouse anti-alpha tubulin: https://www.abcam.com/en-us/products/primary-antibodies/alpha-tubulin-antibody-dm1a-loading-control-ab7291.

Eukaryotic cell lines

Policy information about cell lines and Sex and Gender in Research

Cell line source(s)

The HUVECs used in this study were supplied by Promocell (Cat.No. C-12200) and isolated from single donor Human Umbilical Cord Veins (consistently derived from females). hCMEC/d3 (Cat.No. SCC066; human female-derived) and b.End5 (96091930-1VL; mice-derived, sex unspecified) cell lines were supplied by Merc. Rat TIFs were a Gift from Prof. P.T. Caswell, University of Manchester (sex unspecified).

Authentication

HUVECs, hCMEC/d3 and b.End5 cell lines were authenticated by the suppliers:
(1) https://promocell.com/uk_en/human-umbilical-vein-endothelial-cells-huvec.html
(2) https://www.sigmaaldrich.com/GB/en/product/mm/scc066#product-documentation
(3) https://www.sigmaaldrich.com/GB/en/product/sigma/cb_96091930?srsltid=AfmBOooL8DRNz6mRay8FKFkfC7IPE2ae18aaBXsm1nmHtT3ekbxSAkFb
TIFs were utilised to generate cell-derived matrix and have not been authenticated.

Mycoplasma contamination

All cell lines tested negative for Mycoplasma contamination.

Commonly misidentified lines
(See ICLAC register)

No commonly misidentified cells were used in this study.

Plants

Seed stocks

n/a

Novel plant genotypes

n/a

Authentication

n/a

Flow Cytometry

Plots

Confirm that:

- [ ] The axis labels state the marker and fluorochrome used (e.g. CD4-FITC).
- [ ] The axis scales are clearly visible. Include numbers along axes only for bottom left plot of group (a 'group' is an analysis of identical markers).
- [ ] All plots are contour plots with outliers or pseudocolor plots.
- [ ] A numerical value for number of cells or percentage (with statistics) is provided.

Methodology

Sample preparation	Cultured endothelial cells were trypsinized, pelleted, and washed twice in PBS prior to flow cytometry
Instrument	BD FACS Aria-Fusion
Software	Flow cytometry was exclusively utilised to sort single GFP-expressing endothelial cells into individual wells of 96-well plates for clone expansion. As such, software was not required for flow cytometry data collection/analysis.
Cell population abundance	As mentioned above, flow cytometry was exclusively utilised to collect single GFP-expressing endothelial cells for clone expansion. As such, cell abundance and purity measures were not collected.
Gating strategy	n/a

- [ ] Tick this box to confirm that a figure exemplifying the gating strategy is provided in the Supplementary Information.
